# Red Blood Cell Distribution Width as a Pragmatic Marker for Outcome in Pediatric Critical Illness

**DOI:** 10.1371/journal.pone.0129258

**Published:** 2015-06-09

**Authors:** Alexis L. Ramby, Denise M. Goodman, Eric L. Wald, Scott L. Weiss

**Affiliations:** 1 Division of Critical Care Medicine, Department of Pediatrics, Cincinnati Children’s Hospital Medical Center, Cincinnati, Ohio, United States of America; 2 Division of Critical Care, Department of Pediatrics, Ann & Robert H. Lurie Children’s Hospital of Chicago, Northwestern University Feinberg School of Medicine, Chicago, Illinois, United States of America; 3 Division of Critical Care, Department of Anesthesia and Critical Care Medicine, The Children’s Hospital of Philadelphia, University of Pennsylvania Perelman School of Medicine, Philadelphia, Pennsylvania, United States of America; Bambino Gesù Children's Hospital, ITALY

## Abstract

**Background:**

Red cell distribution width (RDW) is a routine laboratory measure associated with poor outcomes in adult critical illness.

**Objective:**

We determined the utility of RDW as an early pragmatic biomarker for outcome in pediatric critical illness.

**Methods:**

We used multivariable logistic regression to test the association of RDW on the first day of pediatric intensive care unit (PICU) admission with prolonged PICU length of stay (LOS) >48 hours and mortality. The area under the receiver operating characteristic curve (AUROC) for RDW was compared to the Pediatric Index of Mortality (PIM)-2 score.

**Results:**

Over a 13-month period, 596 unique patients had RDW measured on the first day of PICU admission. Sepsis was an effect modifier for LOS >48 hours but not mortality. In sepsis, RDW was not associated with LOS >48 hours. For patients without sepsis, each 1% increase in RDW was associated with 1.17 (95% CI 1.06, 1.30) increased odds of LOS >48 hours. In all patients, RDW was independently associated with PICU mortality (OR 1.25, 95% CI 1.09, 1.43). The AUROC for RDW to predict LOS >48 hours and mortality was 0.61 (95% CI 0.56, 0.66) and 0.65 (95% CI 0.55, 0.75), respectively. Although the AUROC for mortality was comparable to PIM-2 (0.75, 95% CI 0.66, 0.83; p = 0.18), RDW did not increase the discriminative utility when added to PIM-2. Despite the moderate AUROC, RDW <13.4% (upper limit of lower quartile) had 53% risk of LOS >48 hours and 3.3% risk of mortality compared to patients with an RDW >15.7% (lower limit of upper quartile) who had 78% risk of LOS >48 hours and 12.9% risk of mortality (p<0.001 for both outcomes).

**Conclusions:**

Elevated RDW was associated with outcome in pediatric critical illness and provided similar prognostic information as the more complex PIM-2 severity of illness score. Distinct RDW thresholds best discriminate low- versus high-risk patients.

## Introduction

Red cell distribution width (RDW) measures variability in red blood cell size [1] and is a simple, low cost, and widely available measure routinely reported as part of a complete blood count (CBC). Several recent studies suggest that RDW may also be useful as a biomarker of disease severity and clinical outcomes in critically ill patients. An increased RDW is an independent predictor of all-cause mortality in sepsis [2, 3], congestive heart failure [4–6], and adult critical illness [7], and has been shown to improve acute physiology scoring for risk prediction in critically ill adults [8].

Any disease involving red blood cell (RBC) destruction or production can increase variability in RBC size and lead to RDW elevation. In critical illness, the acute systemic inflammatory response resulting from a multitude of underlying etiologies can alter both erythropoiesis and erythrocyte maturation. The resulting acute rise in RDW may therefore reflect the degree of the underlying inflammatory state and provide useful prognostic information about intensity of resource utilization and risk of mortality [5, 9–11]. Similarly, sustained RDW elevation may also be seen in cases of protracted inflammation, as in adults with chronic illnesses [12, 13].

Data on the utility of RDW as a biomarker of clinical outcomes in the pediatric population are more limited. One study demonstrated that preoperative RDW levels were associated with outcomes in children with cardiac disease [14]. However, there are no studies examining RDW as a biomarker in a general pediatric intensive care unit (PICU) population. The characterization of such a readily available biomarker may provide a simple, pragmatic tool to stratify patients by severity of illness and identify those at risk for increased resource utilization and poor outcomes to facilitate focused interventions and triage decisions without additional costs or the need for a novel laboratory assay. We therefore studied the association of RDW at PICU admission with length of stay (LOS) and mortality to determine its potential application as a pragmatic biomarker in the critically ill pediatric population.

## Materials and Methods

We performed a retrospective observational study utilizing an existing database of consecutive patients admitted to an academic 42-bed PICU between May 13, 2009 and June 6, 2010. This study was approved by the Institutional Review Board at Children’s Memorial Hospital (now Ann & Robert H. Lurie Children’s Hospital of Chicago) and a waiver of consent was granted to perform this retrospective chart review of existing data.

### Patient Selection

The medical records of all patients were reviewed for a CBC, including RDW, measured within 24 hours of PICU admission. For patients with more than one PICU admission during the study period, only the initial encounter was included in the analysis and any readmissions were excluded. Although some prior studies of RDW have excluded patients who received recent (between one week to three months) transfusions [2, 15, 16], Purtle et al determined that a RBC transfusion administered as close as 48 hours before RDW measurement did not confound the association of RDW with mortality [17]. Moreover, given the practical challenge to accurately verify blood transfusions given prior to PICU admission in many cases, we chose not to exclude patients with recent blood transfusions to better reflect the utility of RDW as a *pragmatic* biomarker as it would be used in clinical practice.

### Data Collection

Demographic characteristics, admission category, comorbid conditions, laboratory values (RDW, hemoglobin, and RBC mean corpuscular volume [MCV]), PICU LOS, and vital status at PICU discharge were abstracted from the medical records. Patients were categorized into four RDW quartiles based on previously published *a priori* cut-points (RDW < 13.4, 13.4–14.3, 14.4–15.7, and >15.7) [5–7]. The Pediatric Index of Mortality (PIM)-2 score, an internationally validated composite index associated with risk of mortality, was calculated by the institution’s collaborative data capture process with the Virtual PICU Systems (VPS) database (http://www.myvps.org/) and used as the primary measure of severity of illness [18]. Since the most prognostic physiologic and laboratory data have been shown to occur within the first 4 hours of PICU admission, we chose to use PIM-2 over other physiologic scores that consider longer windows of clinical data [19].

We used the term “sepsis” to refer to either severe sepsis and septic shock, which were determined using consensus guidelines, *International Classification of Diseases*, *9* ^*th*^ *Revision*, *Clinical Modification* (ICD-9) codes, and clinical impression as previously described [20, 21]. Patients presenting with “shock” included all those with septic shock, cardiac arrest, hypovolemic shock, cardiogenic shock, anaphylactic shock, and shock not otherwise specified who had documentation of hemodynamic compromise or organ dysfunction requiring fluid resuscitation or vasopressor support. Any patient with a new or pre-existing hematologic or oncologic diagnosis was included in the “hematology/oncology” subgroup (Table 1). As normal values for hemoglobin and hematocrit vary with age, we defined anemia according to the World Health Organization as a hemoglobin lower than 11 g/dL for patients <5 years, lower than 11.5 g/dL for patients 5 to <12 years, lower than 12 g/dL for male patients 12 to <15 years and female patients 12 years and older, and lower than 13 g/dL for male patients 15 years and older [22]^.^

**Table 1:** Patient characteristics.

		RDW Quartile				
Characteristic^1^	All Patients	<13.4%	13.4–14.3%	14.4–15.7%	>15.7%	p-value^2^
Number	596	155	143	151	147	
RDW, %	14.4 (13.3–15.7)	12.8 (12.5–13.1)	13.9 (13.6–14.1)	14.9 (14.6–15.3)	17.5 (16.4–19.3)	<0.001
Age, years	4.4 (1.5–12.9)	8 (3.3–13.1)	3.8 (1.6–12.5)	3.3 (0.7–12.3)	4.4 (1.3–12.9)	<0.001
Sex, n (%)						0.98
Male	316 (53.0)	84 (54.2)	76 (53.1)	80 (53.0)	76 (51.7)	
Female	280 (47.0)	71 (45.8)	67 (46.9)	71 (47.0)	71 (48.3)	
Race, n (%)						0.19
White	256 (43)	81 (52.3)	65 (45.5)	56 (37.1)	54 (36.7)	
Black	117 (19.6)	23 (14.8)	27 (18.9)	32 (21.8)	32 (21.8)	
Hispanic	171 (28.7)	42 (27.1)	40 (28.0)	45 (30.6)	45 (30.6)	
Other	52 (8.7)	9 (5.8)	11 (7.7)	16 (10.9)	16 (10.9)	
PIM-2	1.2 (0.8–4.1)	0.9 (0.3–1.8)	1.4 (0.8–4.3)	1.2 (0.8–4.0)	2.0 (0.9–5.2)	0.001
Hemoglobin, g/dL	11.5 (10.2–13.1)	12.2 (10.8–13.5)	12.2 (10.9–13.4)	11.2 (9.9–13.1)	10.3 (8.6–12.0)	<0.001
MCV, fL	82.0 (78.4–86.5)	82.8 (79.0–86.1)	81.8 (78.9–85.1)	81.9 (78.3–86.6)	82.2 (75.9–90.2)	0.93
Anemia^3^, n (%)	288 (48.3)	56 (36.1)	52 (36.4)	81 (53.6)	99 (67.3)	<0.001
Admit category, n (%)						
Cardiovascular	161 (27.0)	43 (27.7)	52 (36.4)	41 (27.2)	25 (17.0)	0.003
Sepsis	104 (17.4)	10 (6.5)	18 (12.6)	25 (16.6)	51 (34.7)	<0.001
Respiratory	97 (16.3)	28 (18.1)	19 (13.3)	33 (21.9)	17 (11.6)	0.07
Neurologic	77 (12.9)	29 (5.8)	21 (14.7)	15 (9.9)	12 (8.2)	0.03
Airway surgery	35 (5.9)	12 (7.7)	12 (8.4)	7 (4.6)	4 (2.7)	0.12
Gastro/Hepatic	28 (4.7)	9 (5.8)	3 (2.1)	7 (4.6)	9 (6.1)	0.32
Renal	22 (3.7)	3 (1.9)	2 (1.4)	9 (6.0)	8 (5.4)	0.07
Heme/Onc	21 (3.5)	3 (1.9)	1 (0.7)	5 (3.3)	12 (8.2)	0.005
Orthopedic	20 (3.4)	7 (4.5)	8 (5.6)	3 (2.0)	2 (1.4)	0.15
Trauma	8 (1.3)	5 (3.2)	1 (0.7)	0 (0.0)	2 (1.4)	0.08
Other	23 (3.9)	6 (3.9)	6 (4.2)	6 (4.0)	5 (3.4)	0.99
Comorbid conditions, n (%)^4^						
None	398 (66.8)	123 (79.4)	115 (80.4)	95 (62.9)	65 (44.2)	<0.001
Heme/Oncology	60 (10.1)	9 (5.8)	8 (5.6)	16 (10.6)	27 (18.4)	0.001
Cardiovascular	34 (5.7)	3 (1.9)	6 (4.2)	14 (9.3)	11 (7.5)	0.02
Respiratory	36 (6.0)	7 (4.5)	6 (4.2)	8 (5.3)	15 (10.2)	0.14
Gastro/Hepatic	28 (4.7)	2 (1.3)	5 (3.5)	8 (5.3)	13 (8.8)	0.02
Neurologic	15 (2.5)	8 (5.2)	0 (0.0)	3 (2.0)	4 (2.7)	0.03
Renal	7 (1.2)	0 (0.0)	0 (0.0)	3 (2.0)	4 (2.7)	0.13
Other	18 (3.0)	3 (1.9)	2 (1.4)	5 (3.3)	7 (4.8)	0.20
Heme/Onc, n (%)^5^	81 (13.6)	12 (7.7)	9 (6.3)	21 (13.9)	39 (26.5)	<0.001
Surgical, n (%)	230 (38.6)	66 (42.6)	79 (55.2)	54 (35.8)	31 (21.1)	<0.001
Sepsis, n (%)	111 (18.6)	12 (7.7)	19 (13.3)	28 (18.5)	52 (35.4)	<0.001
Shock, n (%)	105 (17.6)	13 (8.4)	16 (11.2)	26 (17.2)	50 (34.0)	<0.001
Outcomes						
PICU LOS, days	4 (2–9)	3 (1–5)	3 (2–8)	5 (2–11)	5 (3–13)	<0.001
PICU LOS >48 hrs, n (%)	412 (69.1)	83 (53.5)	100 (69.9)	114 (75.5)	115 (78.2)	<0.001
PICU mortality, n (%)	39 (6.5)	5 (3.2)	7 (4.9)	8 (5.3)	19 (12.9)	<0.001

RDW, red blood cell distribution width; MCV, mean corpuscular volume; PIM-2, Pediatric Index of Mortality-2

^1^Data expressed as median (interquartile range) otherwise specified. IQR, interquartile range

^2^Statistical comparisons across RDW groups

^3^Anemia was defined as by World Health Organization criteria [22]

^4^Comorbid conditions do not include the primary admission category.

^5^Includes all patients with a primary or comorbid hematologic/oncologic condition.

### Laboratory Measurements

RDW was measured as part of the routine CBC using a Siemens Advia 2120 Hematology Analyzer according to the formula:

RDW=(CoefficientofVariabilityofRBC÷meanMCV)×100

Periodic comparisons between two routinely used identical analyzers were performed as part of the clinical laboratory accreditation requirements. The reference range for RDW in our laboratory is 12.5–16.0%.

### Outcomes

PICU LOS is a commonly used clinical endpoint reflecting both severity of illness and resource utilization [23]. However, because LOS is influenced by a variety of clinical and logistic factors that may not be completely apparent in a retrospective chart review, we *a priori* decided to analyze LOS as a dichotomous outcome of less than or equal to 48 hours versus greater than 48 hours in order to determine if admission RDW was increased in those patients requiring more intensive PICU resource utilization (i.e., LOS >48 hours). We chose the cut-point of 48 hours because a prior study of 52,791 admissions to 54 PICUs in the United States reported a median LOS of 1.4 (interquartile range 0.8–3) days supporting that the majority of pediatric patients require <48 hours of PICU admission [23]. We therefore used PICU LOS >48 hours to indicate greater severity of illness, increased resource utilization, or both. The secondary outcome measure was all-cause PICU mortality.

### Statistical Analysis

Statistical analysis was performed using STATA (Version 12.1, College Station, TX). Continuous variables were non-normally distributed and presented as medians with interquartile range (IQR) and compared using the Kruskal-Wallis test for non-normally distributed data. Categorical data are presented as proportions and compared using chi-squared or Fisher’s exact tests as appropriate. We used multivariable logistic regression to assess potential confounding and effect modification of select clinical variables on the association of RDW with outcomes. We evaluated for co-linearity of variables using Spearman’s correlation coefficient (ρ). For multivariable models, RDW was entered as a continuous variable. Covariates were selected based on biological plausibility, data availability, and prior studies [2–12]. Potential confounders were included in the model one at a time; those that changed the base model OR by 10% or greater were considered to be true confounders [24]. Effect modification was considered present if the interaction of the variable with RDW achieved a p-value ≤0.20. We predetermined that all multivariable models would be adjusted for age, hemoglobin, and PIM-2 even if these variables did not reach the threshold for confounding or effect modification. Since PIM-2 scores were available for only 64% of the study population, we used multiple imputations with 20 iterations to address missing PIM-2 scores under the “missing at random” assumption [25] because excluding these patients was felt to introduce bias [26]. Multivariable analyses were performed using imputed data, and both unadjusted and adjusted odds ratios (ORs) with 95% confidence intervals are presented. The area under the receive operating characteristic curve (AUROC) was used to determine the discrimination of RDW for LOS >48 hours and mortality and to define optimal cut-points for sensitivity, specificity, and positive and negative predictive values (PPV, NPV). The incremental benefit of adding RDW to the PIM-2 score to predict PICU mortality was determined by comparing the AUROC of PIM-2 alone with that of a multivariable model including both PIM-2 and RDW. Comparison of AUROCs was performed by generating linear predictions following separate logistic regression models with PICU mortality as the outcome and either RDW alone or RDW and PIM-2 as independent variables [27]. The AUROC generated from these two linear predictors were then compared as previously described [28]. P-values <0.05 were considered statistically significant.

## Results

**Fig 1:**
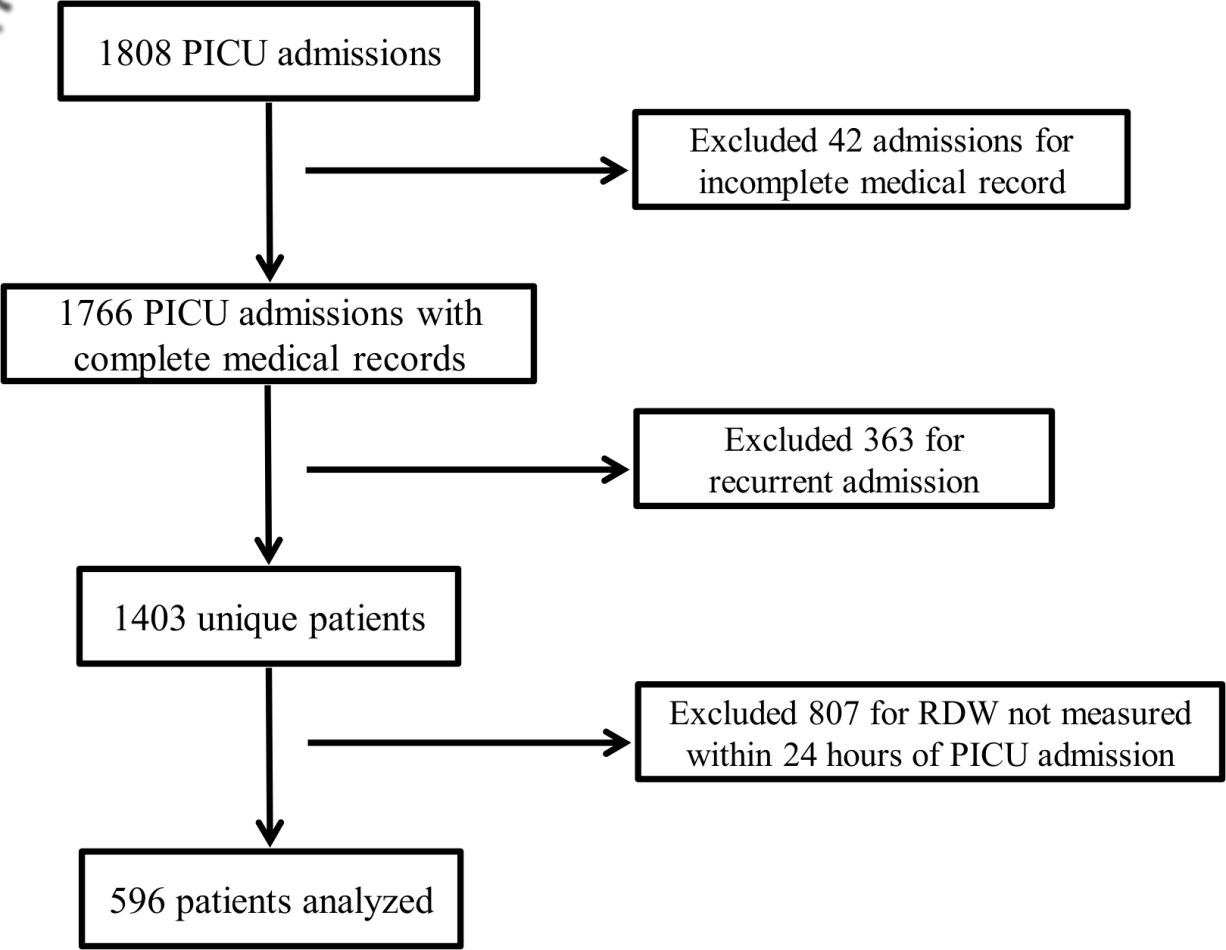
Patient Selection. Flow diagram of patient selection for the study population.

Of the 1808 total PICU admissions over the study period, 42 were excluded for incomplete medical record and 363 were excluded for recurrent admission. Of the 1403 unique patients, 596 (42%) had an RDW measured within 24 hours of PICU admission and were included in the analysis (Fig 1). Baseline characteristics of the study population are presented in Table 1. As expected based on prior studies, approximately 25% of patients were distributed within each RDW group. Four hundred twenty (70%) patients were admitted directly to the study institution and 176 (30%) were transferred from a referring hospital.

The median RDW for all patients was 14.4% (IQR 13.3–15.7%). Age, hemoglobin, admit category, comorbid conditions, and proportion with anemia, hematologic/oncologic illness, sepsis, shock, and surgical disease differed significantly across RDW groups (Table 1). As expected, RDW was inversely correlated with hemoglobin, though the magnitude of this association was weak (ρ = -0.30, p<0.001). RDW was not correlated with MCV (ρ = -0.01, p = 0.74).

The proportion of patients with PICU LOS >48 hours increased in a step-wise fashion for each successive RDW group (Table 1). In the multivariable analysis of RDW with PICU LOS >48 hours, sepsis at PICU admission emerged as an important effect modifier and data are therefore presented separately for patients with (n = 111) and without (n = 485) sepsis. None of the covariates listed in Table 1 met the threshold for confounding, though age, hemoglobin, and PIM-2 were forced into the final model as planned. Since RDW and hemoglobin were weakly correlated, we confirmed that including hemoglobin as a covariate did not adversely impact the overall model. For patients with sepsis, RDW was not associated with PICU LOS >48 hours. For patients without sepsis, each 1% increase in RDW was associated with 1.17 (95% CI 1.06, 1.30) increased odds of PICU LOS >48 hours (Table 2).

**Table 2:** Multivariable association of RDW with PICU LOS >48 hours.

Variable	Adjusted OR (95% CI)^1^	p-value	Adjusted OR (95% CI)^1^	p-value
	Sepsis present (n = 111)		Sepsis not present (n = 485)	
RDW	0.91 (0.73, 1.13)	0.39	1.17 (1.06, 1.30)	0.003
Age	1.05 (0.96, 1.15)	0.32	0.95 (0.92, 0.98)	0.001
Hemoglobin	0.81 (0.64, 1.04)	0.10	1.12 (1.02, 1.22)	0.01
PIM-2	1.16 (0.98, 1.38)	0.09	1.02 (0.99, 1.05)	0.13

OR, odds ratio; CI, confidence interval; RDW, red blood cell distribution width; PICU, pediatric intensive care unit; PIM-2, pediatric risk of mortality-2

^1^Analyses adjusted for the other variables listed

Overall PICU mortality was 6.5%. There was a significant increase in mortality across RDW quartiles, with mortality rising from 3.2% in the lowest to 12.9% in the highest RDW quartile (p<0.001; Table 1). None of the covariates listed in Table 1 met the threshold for confounding or effect modification (although PIM-2 was itself independently associated with PICU mortality). After controlling for age, hemoglobin, and PIM-2, each 1% increase in RDW increased the odds of PICU mortality by 1.20 (95% CI 1.07, 1.35; Table 3).

**Table 3:** Multivariable association of RDW with PICU mortality.

Variable	Adjusted OR (95% CI)^1^	p-value
RDW	1.20 (1.07, 1.35)	0.002
Age	1.01 (0.96, 1.08)	0.66
Hemoglobin	0.97 (0.84, 1.11)	0.61
PIM-2	1.05 (1.03, 1.08)	<0.001

OR, odds ratio; CI, confidence interval; RDW, red blood cell distribution width; PICU, pediatric intensive care unit; PIM-2, pediatric risk of mortality-2

^1^Analyses adjusted for the other variables listed

**Fig 2:**
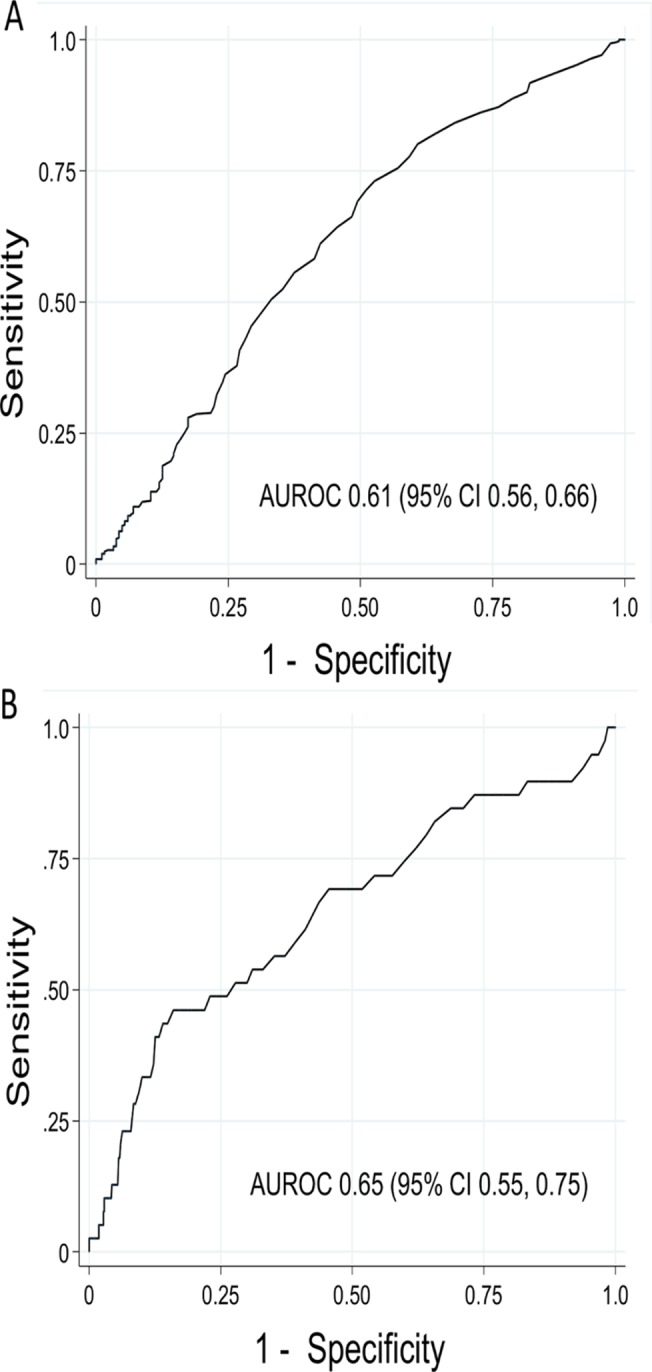
Receiver operating characteristic curves for RDW and outcomes. Receiver operating characteristic (ROC) curves for RDW measured within 24 hours of PICU admission to predict PICU LOS >48 hours (a) and all-cause PICU mortality (b). AUROC is the area under the ROC curve.

RDW had marginal discriminative power for LOS >48 hours (AUROC 0.61, 95% CI 0.56, 0.66) and for mortality (AUROC 0.65, 95% CI 0.55, 0.75), with the ROC curves shown in the appendix (Fig 2A and 2B, respectively). The optimal RDW cut-point for LOS >48 hours was ≥13.9%, which yielded sensitivity 69% (95% CI 64, 74%), specificity 51% (95% CI 43, 58%), PPV 76% (95% CI 71, 80%), and NPV 42% (95% CI 36, 49%). The optimal RDW cut-point for mortality was ≥14.5%, which yielded sensitivity 69% (95% CI 52, 83%), specificity 54% (95% CI 50, 59%), PPV 10% (95% CI 6, 14%), and NPV 96% (95% CI 93, 98%). However, when distinct thresholds were considered to identify low-risk versus high-risk groups, patients with an RDW <13.4% (upper limit of lower quartile) had a 53% risk of LOS >48 hours and 3.3% risk of mortality compared to patients with an RDW >15.7% (lower limit of upper quartile) who had a 78% risk of LOS >48 hours and 12.9% risk of mortality (p<0.001 for both outcomes). Therefore, an RDW <13.4% yielded negative predictive values of 47% (95% CI 39, 55%) and 96.7% (95% CI 92.6, 98.9%) for PICU LOS >48 hours and mortality, respectively, compared to an RDW >15.7% which yielded positive predictive values of 78% (95% CI 71, 85%) and 12.9% (95% CI 8.0, 19.4%) for PICU LOS >48 hours and mortality, respectively.

**Fig 3:**
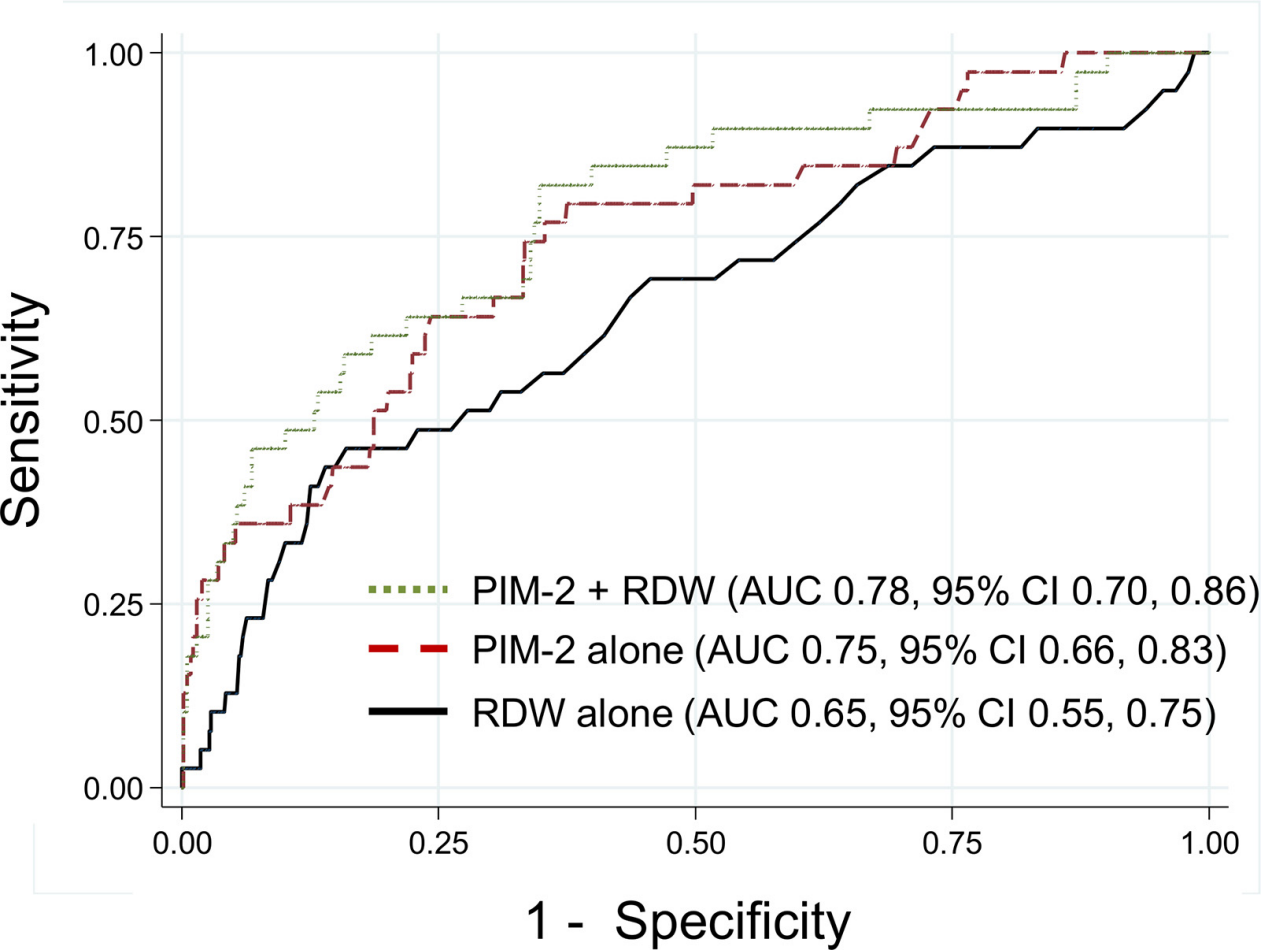
Comparison of receiver operating characteristic curves for RDW and PIM-2. Receiver operating characteristic (ROC) curves for RDW (solid black line), PIM-2 (dashed maroon line) and the combination of PIM-2 and RDW (dotted green line) to predict all-cause PICU mortality. There was no difference between the AUROC for RDW and PIM-2 (p = 0.18). The addition of RDW to PIM-2 did not increase the AUROC compared to PIM-2 alone (p = 0.49). AUROC is the area under the ROC curve.

The AUROC for RDW was comparable to the well-validated PIM-2 score to predict mortality (AUROC 0.65, 95% CI 0.55, 0.75 versus 0.75, 95% CI 0.66, 0.83; p = 0.18). However, as shown in Fig 3, the addition of RDW did not significantly increase the discriminative ability of the PIM-2 score alone (AUROC for PIM-2 combined with RDW: 0.78, 95% CI 0.70, 0.86; p = 0.49).

To determine the utility of RDW for critically ill children with a known onset of critical illness, we performed a separate analysis including only the 420 patients admitted directly to the study institution without prior care at a referral hospital. Patients admitted directly to the study institution had a slightly lower severity of illness and were more likely to be admitted for a cardiovascular, respiratory, or post-operative problem but there were not differences in outcomes compared to patients transferred from a referring hospital (S1 Table). For patients with sepsis, RDW was not associated with PICU LOS >48 hours after controlling for age, hemoglobin, and PIM-2. For patients without sepsis, each 1% increase in RDW was independently associated with 1.18 (95% CI 1.04, 1.35) increased odds of PICU LOS >48 hours (S2 Table). Each 1% increase in RDW increased the odds of PICU mortality by 1.24 (95% CI 1.06, 1.45; S3 Table). In this subset of patients, RDW achieved similar discriminative power as for the full cohort for both LOS >48 hours (AUROC 0.62, 95% CI 0.56, 0.68) and mortality (AUROC 0.64, 95% CI 0.50, 0.77; S1 Fig).

## Discussion

We found that RDW measured within 24 hours of PICU admission was independently associated with length of intensive care unit stay >48 hours for patients without (but not with) sepsis and with mortality in all patients. Although the test characteristics of RDW to predict PICU LOS >48 hours and mortality achieved only moderate clinical utility when single cut-points were used in the ROC analysis, the discriminative utility of RDW improved when different thresholds were considered to identify low- versus high-risk groups such that an RDW <13.4% achieved a high (96.7%) negative predictive value for mortality. Most importantly, a single measurement of RDW, with its low cost and widespread availability, performed nearly as well as the more complex PIM-2 score to predict PICU mortality.

RDW has shown utility as a biomarker associated with mortality in adult patients with both chronic illness (congestive heart failure, cancer, pulmonary hypertension, arteriosclerosis) and acute illness (pneumonia, sepsis, blood stream infections, stroke) [2–9]. There are limited data testing the utility of RDW in critically ill children [12]. Our data demonstrate that RDW at the time of PICU admission may help to alert PICU clinicians to a subgroup of patients within the general, critically ill pediatric population who are at risk for adverse outcomes. Early identification of these at-risk patients may provide an opportunity to intervene and thereby improve outcomes and optimize resource utilization.

The most attractive properties of RDW as a pragmatic clinical biomarker are its relative low cost and near universal availability compared to other proposed biomarkers in this population. Although the AUROC for RDW achieved only modest clinical utility overall, this statistical metric fails to fully capture whether different cut-points may be useful to define variable risk categories. For example, the upper limit of lower RDW quartile (<13.4%) achieved a NPV of 96.7% to rule out mortality, which was comparable to the 97% NPV reported in a validation study of a multi-biomarker algorithm generated using a sophisticated genome-wide expression algorithm in pediatric septic shock [29]. Objective prognostic scores that are based on routine clinical and laboratory data, such as PIM-2 or Pediatric Risk of Mortality (PRISM) scores, may also be useful to guide communication, triage, and management decisions for critically ill patients but these scores are complex to calculate and experts have cautioned against using these to predict outcomes for individual patients [30, 31].

RDW is known to be elevated in states of ineffective red cell production and increased red cell destruction, which are a common feature in a variety of infectious and inflammatory conditions [1, 10, 11]. An association between increasing RDW and elevated levels of acute phase reactants including erythrocyte sedimentation rate, high sensitivity C-reactive protein, and interleukin-6 has been demonstrated in adults, suggesting that RDW may be elevated in the setting of acute inflammatory states secondary to rapid red blood cell destruction or blunted erythropoiesis [5, 7, 10, 11]. However, prior studies have revealed that increased RDW remains predictive of outcomes after controlling for known inflammatory markers, indicating that inflammation alone cannot entirely explain the pathophysiologic processes leading to RDW elevation in critical illness [2, 5, 7]. In our study, patients with the highest RDW were more likely to present with infection, sepsis, and shock. Although this implies a causative role for inflammation to increase RDW, we lacked specific measures to determine the extent to which inflammation modified the association of RDW with LOS or mortality in our patients. A key point, however, is that RDW is most likely to be a marker of an underlying pathophysiological process (i.e., inflammation, impaired erythropoiesis, or bone marrow dysfunction) rather than itself being a cause of adverse clinical outcomes.

Adult studies have demonstrated that RDW remains an independent predictor of mortality after controlling for recent blood transfusions [2–9]. In our study, we did not control for transfusions prior to RDW collection because our objective was to determine if RDW, regardless of underlying cause of elevation, was predictive of outcome. Furthermore, the inclusion of patients regardless of recent transfusion improves the generalizability of our findings. RDW is also elevated in several types of anemia, so it could be asserted that we are simply describing unrecognized anemia of chronic illness or iron-deficiency anemia in critically ill children. However, the persistent association of RDW with both mortality in all patients and PICU LOS in the non-septic population after controlling for hemoglobin suggests that anemia cannot account entirely for our findings.

Our study has several limitations. First, since this was a secondary analysis of an existing database, we were not able to assess all possible confounding variables, such as measures of iron deficiency, markers of nutritional status, or biomarkers of inflammation. In addition, while using VPS ensured that PIM-2 scores were calculated in a validated manner, only two-thirds of patients had an accessible PIM-2 score and the more contemporary PIM-3 severity of illness score, which may achieved a higher AUROC for PICU mortality than PIM-2 [32], was not available for analysis. Second, only 42% of the eligible patients had an RDW available in the first 24 hours of PICU admission, which may be a source of selection bias. As a tertiary referral center, many patients are transferred to our center and we did not have access to laboratory evaluations performed at referring centers. Third, even though we chose not to control for RBC transfusions prior to RDW measurement, we acknowledge that transfusion can alter the RDW measurement. However, using the first RDW available following PICU admission minimized the likelihood that a patient would have received a PRBC transfusion immediately before this measurement. Fourth, the lack of an association between RDW and LOS in septic patients may be attributable to the relatively small number of septic patients. In addition, the biologic heterogeneity inherent within the septic subgroup may diminish the discriminating potential of RDW in this group. Finally, due to the retrospective nature of this study, we were not able to test the clinical utility of RDW in combination with other laboratory biomarkers of inflammation and poor clinical outcomes.

## Conclusions

RDW measured within 24 hours of PICU admission was independently associated with PICU LOS >48 hours in non-septic patients and mortality in a general PICU population. While the test characteristics of a single RDW cut-point achieved only moderate clinical utility overall, discrimination of low- versus high-risk patients improved when quartile-based thresholds were considered and RDW alone performed similarly to the more complex PIM-2 illness severity scoring system. The low cost and near universal availability of RDW enhance its pragmatic value as an adjunctive biomarker in critically ill children. Our data support the need to perform prospective longitudinal studies in larger populations to better determine the utility of RDW to augment other clinical data for decision-making in the PICU.

## Supporting Information

S1 Fig Receiver operating characteristic curves for RDW and outcomes for subset of patients admitted directly to the study institution. Receiver operating characteristic (ROC) curves for RDW measured within 24 hours of PICU admission to predict PICU LOS >48 hours (a) and all-cause PICU mortality (b). AUROC is the area under the ROC curve.
(TIFF)

S1 Table Characteristics of patients admitted directly to the study institution and patients transferred from a referring hospital (DOCX)

S2 Table Multivariable association of RDW with PICU LOS >48 hours for the subset of patients admitted directly to the study institution (DOCX)

S3 Table Multivariable association of RDW with PICU mortality for the subset of patients admitted directly to the study institution (DOCX)

S4 Table Study dataset. The primary data for this study are available in S4 Table.
(XLS)
