# Supplementary figures and images for: Red Blood Cell Distribution Width as a Pragmatic Marker for Outcome in Pediatric Critical Illness
Source: PLoS One. 2015 Jun 9;10(6):e0129258. doi: 10.1371/journal.pone.0129258 (PMC4461244; doi:10.1371/journal.pone.0129258)

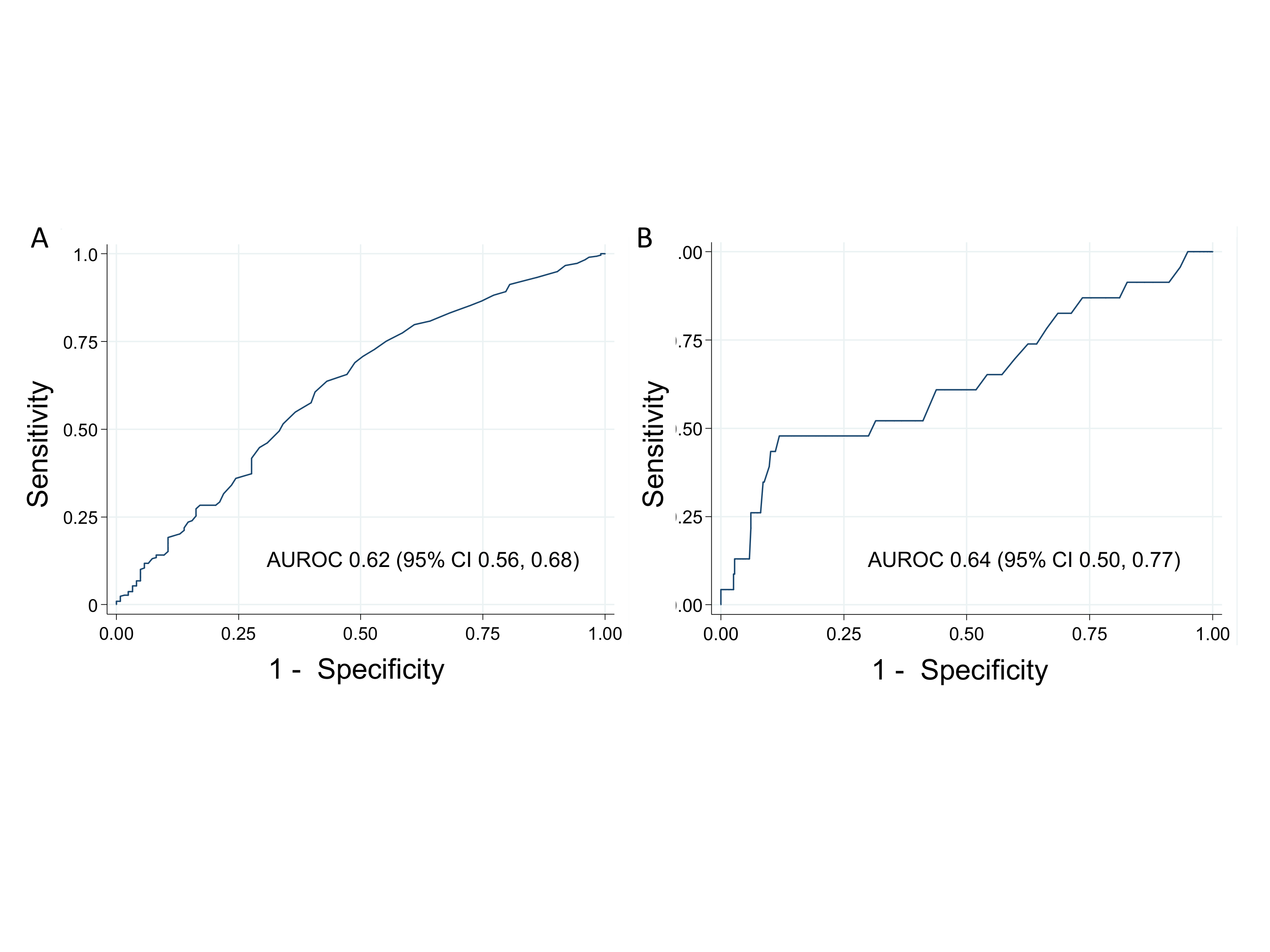

Supplement: S1 Fig — Receiver operating characteristic (ROC) curves for RDW measured within 24 hours of PICU admission to predict PICU LOS >48 hours (a) and all-cause PICU mortality (b). AUROC is the area under the ROC curve. (TIFF) [file pone.0129258.s001.tiff]
