# Supplementary material for: Red Blood Cell Distribution Width as a Pragmatic Marker for Outcome in Pediatric Critical Illness
Source: PLoS One. 2015 Jun 9;10(6):e0129258. doi: 10.1371/journal.pone.0129258 (PMC4461244; doi:10.1371/journal.pone.0129258)
Supplement: S1 Table — (DOCX) [file pone.0129258.s002.docx]

S1 Table: Characteristics of patients admitted directly to the study institution and patients transferred from a referring hospital

| **Characteristic^1^** | | **Direct Admission** | **Transfer from Referring Hospital** | **p-value^2^** |
| --- | --- | --- | --- | --- |
| Number | | 420 | 176 |  |
| RDW, % | | 14.3 (13.3 (15.7) | 14.4 (13.3-15.9) | 0.67 |
| Age, years | | 4.5 (1.8-12.8) | 4.4 (1.2-13.0) | 0.52 |
| Sex, n (%) | |  |  | 0.34 |
|  | Male | 228 (54) | 88 (50) |  |
|  | Female | 192 (46) | 88 (50 |  |
| Race, n (%) | |  |  | 0.11 |
|  | White | 188 (45) | 68 (39) |  |
|  | Black | 72 (17) | 45 (26) |  |
|  | Hispanic | 124 (30) | 47 (27) |  |
|  | Other | 36 (9) | 16 (9) |  |
| PIM-2 | | 1.1 (0.6-3.3)^6^ | 2.0 (0.9-5.8)^6^ | <0.001 |
| Hemoglobin, g/dL | | 11.6 (10.2-13.1) | 11.3 (10.0-13.2) | 0.52 |
| MCV, fL | | 82.1 (78.6-86.4) | 81.9 (78.0-87.1) | 0.90 |
| Anemia^3^, n (%) | | 200 (48) | 88 (50) | 0.60 |
| Admit category, n (%) | |  |  |  |
|  | Cardiovascular | 128 (30) | 33 (19) | 0.003 |
|  | Sepsis | 72 (17) | 32 (18) | 0.76 |
|  | Respiratory | 57 (14) | 40 (23) | 0.006 |
|  | Neurologic | 47 (11) | 30 (17) | 0.05 |
|  | Airway surgery | 27 (6) | 8 (5) | 0.45 |
|  | Gastro/Hepatic | 22 (5) | 6 (3) | 0.34 |
|  | Renal | 15 (4) | 7 (4) | 0.81 |
|  | Heme/Onc | 13 (3) | 8 (5) | 0.47 |
|  | Orthopedic | 18 (4) | 2 (1) | 0.08 |
|  | Trauma | 7 (2) | 1 (1) | 0.45 |
|  | Other | 14 (3) | 9 (5) | 0.35 |
| Comorbid conditions, n (%)^4^ | |  |  |  |
|  | None | 284 (68) | 114 (65) | 0.50 |
|  | Heme/Oncology | 44 (11) | 16 (9) | 0.61 |
|  | Cardiovascular | 19 (5) | 15 (9) | 0.06 |
|  | Respiratory | 23 (5) | 13 (7) | 0.37 |
|  | Gastro/Hepatic | 25 (6) | 3 (2) | 0.03 |
|  | Neurologic | 8 (2) | 7 (4) | 0.16 |
|  | Renal | 3 (1) | 4 (2) | 0.20 |
|  | Other | 14 (3) | 4 (2) | 0.60 |
| Heme/Onc, n (%)^5^ | | 50 (12) | 19 (11) | 0.70 |
| Surgical, n (%) | | 206 (49) | 24 (14) | <0.001 |
| Sepsis, n (%) | | 76 (18) | 35 (20) | 0.61 |
| Shock, n (%) | | 72 (17) | 33 (19) | 0.64 |
| Outcomes | |  |  |  |
|  | PICU LOS, days | 4 (2-9) | 4 (2-9) | 0.98 |
|  | PICU LOS >48 hrs, n (%) | 297 (71) | 115 (65) | 0.20 |
|  | PICU mortality, n (%) | 23 (6) | 16 (9) | 0.10 |

RDW, red blood cell distribution width; MCV, mean corpuscular volume; PIM-2, Pediatric Index of Mortality-2

^1^Data expressed as median (interquartile range) otherwise specified. IQR, interquartile range

^2^Statistical comparisons across RDW groups
^3^Anemia was defined as by World Health Organization criteria [22]

^4^Comorbid conditions do not include the primary admission category

^5^Includes all patients with a primary or comorbid hematologic/oncologic condition

^6^PIM-2 scores were available for 273 (65%) of patients admitted directly to the study institution and for 107 (61%) of patients transferred from a referring hospital
