# Supplementary material for: Red Blood Cell Distribution Width as a Pragmatic Marker for Outcome in Pediatric Critical Illness
Source: PLoS One. 2015 Jun 9;10(6):e0129258. doi: 10.1371/journal.pone.0129258 (PMC4461244; doi:10.1371/journal.pone.0129258)
Supplement: S2 Table — (DOCX) [file pone.0129258.s003.docx]

S2 Table: Multivariable association of RDW with PICU LOS >48 hours for the subset of patients admitted directly to the study institution

| **Variable** | **Adjusted OR (95% CI)^1^** | **p-value** |  | **Adjusted OR (95% CI)^1^** | **p-value** |
| --- | --- | --- | --- | --- | --- |
|  | Sepsis present (n=76) | |  | Sepsis not present (n=344) | |
| RDW | 1.05 (0.82, 1.35) | 0.69 |  | 1.18 (1.04, 1.35) | 0.01 |
| Age | 1.0 (0.88, 1.11) | 0.97 |  | 0.94 (0.90, 0.98) | 0.002 |
| Hemoglobin | 1.05 (0.77, 1.44) | 0.75 |  | 1.08 (0.96, 1.22) | 0.19 |
| PIM-2 | 1.17 (0.92, 1.50) | 0.19 |  | 1.02 (0.98, 1.07) | 0.40 |

OR, odds ratio; CI, confidence interval; RDW, red blood cell distribution width; PICU, pediatric intensive care unit; PIM-2, pediatric risk of mortality-2

^1^Analyses adjusted for the other variables listed
