# Supplementary material for: Red Blood Cell Distribution Width as a Pragmatic Marker for Outcome in Pediatric Critical Illness
Source: PLoS One. 2015 Jun 9;10(6):e0129258. doi: 10.1371/journal.pone.0129258 (PMC4461244; doi:10.1371/journal.pone.0129258)
Supplement: S3 Table — (DOCX) [file pone.0129258.s004.docx]

S3 Table: Multivariable association of RDW with PICU mortality for the subset of patients admitted directly to the study institution

| **Variable** | **Adjusted OR (95% CI)^1^** | **p-value** |
| --- | --- | --- |
| RDW | 1.24 (1.06, 1.45) | 0.007 |
| Age | 1.01 (0.94, 1.09) | 0.74 |
| Hemoglobin | 0.86 (0.69, 1.06) | 0.16 |
| PIM-2 | 1.10 (1.05, 1.16) | <0.001 |

OR, odds ratio; CI, confidence interval; RDW, red blood cell distribution width; PICU, pediatric intensive care unit; PIM-2, pediatric risk of mortality-2

^1^Analyses adjusted for the other variables listed
